# Resource utilization in the sub-sectors of the textile industry: opportunities for sustainability

**DOI:** 10.1007/s11356-024-32768-2

**Published:** 2024-03-12

**Authors:** Alperen Kır, Emrah Ozturk, Ulku Yetis, Mehmet Kitis

**Affiliations:** 1 Department of Environmental Engineering, Suleyman Demirel University, 32200 Isparta, Turkey; 2 Department of Environmental Protection Technologies, Isparta University of Applied Sciences, 32500 Isparta, Turkey; 3 Department of Environmental Engineering, Middle East Technical University, 06800 Ankara, Turkey

**Keywords:** Chemical, Cleaner production, Dyestuff, Electricity, Steam, Textile, Water

## Abstract

It was aimed to determine the specific resource use and reduction potential profiles in various textile sub-sectors (cotton woven fabric dyeing-finishing, wool woven fabric dyeing-finishing, synthetic woven fabric dyeing-finishing, cotton knitted fabric, synthetic knit fabric dyeing-finishing, non-woven fabric, dyeing-finishing of knitted fabric). The main focus was to elucidate opportunities for sustainability in terms of decreasing resource utilization in the textile sector. On-site surveys and detailed data collection studies were carried out at 150 textile facilities. Average specific values for water, auxiliary chemicals, dyestuff, electricity, and steam consumptions, and related reduction potentials were calculated and compared within facilities and sub-sectors. The minimum specific resource consumption values reported in the Best Available Techniques Reference Document (BREF) for the textile industry and data of similar facilities from the literature were evaluated and used. A detailed environmental performance profile of the Turkish textile sector in terms of resource usage and reduction potential was generated. The highest specific water consumption was found in the wool-woven fabric sub-sector (345 ± 262 L/kg product). Although the specific auxiliary chemical consumption shows similarities within sub-sectors, the highest specific auxiliary chemical consumption (397 ± 237 g/kg product) was found in the synthetic woven fabric sub-sector. The sub-sector with the highest specific dyestuff consumption (30 ± 13 g/kg product) was the cotton knitted fabric sub-sector. The wool woven fabric industry had the highest specific electricity (7 ± 5.3 kWh/kg product) and steam (20 ± 11 kg steam/kg product) consumption. In addition, for all the studied sub-sectors country-wide, the lowest and highest reduction potentials in resource uses were 18 ± 15% and 73 ± 13%, respectively, suggesting a need for major full-scale implementations of cleaner production for enhancing sustainability in the textile industry.

## Introduction

Today, more than 150 countries are textile suppliers on a global scale (Luoma et al. 2022). Textile and clothing industry (T&C) is the second largest industry in the manufacturing sector (Okafor et al. 2021). Turkey has a share of approximately 3.7% in the global textile trade by exporting approximately 11.7 billion dollars of textiles according to the World Trade Statistical Review 2020 report of the World Trade Organization (WTO) (World Trade Organization (WTO), 2021). Turkey is the world’s fifth largest textile supplier in terms of textile exports (World Trade Organization (WTO), 2021). In addition, Turkey is the second largest textile supplier in Europe after China (Halife 2022). In Turkey, the textile and garment sector is one of the locomotive sectors of the manufacturing industry (United Nations Development Program (UNDP), 2021). The textile industry in Turkey has a heterogeneous structure consisting of many sub-sectors (Tregenna and Andreoni 2020). In addition, Europe’s largest textile facilities in terms of production capacity in various sub-sectors are also located in Turkey.

In Turkey, which is in the process of full membership to the European Union (EU), the process of harmonization of environmental legislation with EU directives is still ongoing. One of the most important directives evaluated in the harmonization process is the Industrial Emissions Directive (IED-2010). The first step taken in Turkey was the publication of Communiqué on Integrated Pollution Prevention and Control in the Textile Sector in 2011 (Turkish Ministry of Environment and Urbanization (TMEU) 2021) in line with the Best Available Techniques Reference Document (BREF) for the textile industry (TXT BREF) published by European Commission (EC) (Roth et al. 2023). The Integrated Pollution Prevention and Control Regulation was published as a Draft in 2018, and it is planned to be implemented in 2024 (Turkish Ministry of Environment and Urbanization (TMEU) 2021). The Turkish Ministry of Environment, Urbanization, and Climate Change prepared Communiqués and BREFs for various industrial sectors. For the textile sector, which is among these sectors, a project (BESTÜ Project) was carried out to revise the current communiqué that entered into effect in 2011 and to prepare BREF documents for the textile sector. Within this project, in which the authors were also involved, various textile sub-sectors in Turkey were studied and resource usage (raw material, water, chemical, and energy) and environmental performances were investigated. BESTÜ project was carried out with the cooperation of the Ministry, Universities, Textile Associations, and textile facilities. On-site investigations, comprehensive data collection, and detailed survey studies were carried out in 150 textile facilities with the participation of facility technical personnel. The main purpose was to take a general picture of the industry in terms of existing resource uses and determination of reduction potentials with respect to those indicated in the TXT BREF. In other words, the current situation of cleaner production performance was determined country-wide in the textile industry.

This paper was prepared using some of the industrial data obtained from the BESTÜ Project. Specific resource consumptions and reduction potentials were calculated for each textile sub-sector in Turkey. The studied sub-sectors were as follows: cotton woven fabric dyeing-finishing, wool woven fabric dyeing-finishing, synthetic woven fabric dyeing-finishing, cotton knitted fabric, synthetic knit fabric dyeing-finishing, non-woven fabric, and dyeing-finishing of knitted fabric. The performances of each facility in each sub-sector were compared side by side, and environmental performance profiles were generated. As noted before, the main focus was to evaluate the current sustainability and cleaner production performance of all the textile sub-sectors. While doing this task, opportunities for improved cleaner production in the sector were also determined. Therefore, in addition to its national importance in terms of contributing to the process of establishing IED-related legislations in Turkey, this study will also aid global textile sector in terms of benchmarking and implementing full-scale cleaner production measures. According to the best knowledge of the authors, apart from the TXT BREF, there has been no specific study in the literature that examined textile sub-sectors country-wide in detail in terms of their resource uses and determination of reduction potentials using real data from many facilities. We believe that specific resource use values obtained in this study from 150 textile facilities (44 of them are reported in this paper due to project agreements) will aid the global textile sector, researchers, and decision-makers in terms of benchmarking and comparison purposes. Furthermore, this original paper may also be beneficial to other global industries since they may use the employed methodology in this work and determine sustainability opportunities in their sectors.

## Methodology

The number of facilities, production capacities, fields of activity, and locations in the textile sub-sectors in Turkey were determined by examining non-public official data. A detailed inventory of the Turkish textile sub-sector was prepared. It was found that major textile sub-sectors were woven and knitted fabric dyeing-finishing (at an average of 40%) and non-woven (at an average of 19%). From these data, it was decided to carry out data collection studies in the sub-sectors of cotton woven fabric, wool woven fabric, synthetic woven fabric, cotton knitted fabric, synthetic knitted fabric, and non-woven fabric production. The locations of the textile sub-sectors in Turkey were also determined. It was determined that the detailed sub-sectors decided to be examined in this study are concentrated in the provinces of Denizli, Tekirdag, Istanbul, Gaziantep, Kahramanmaras, and Bursa. Therefore, data collection studies were carried out in these provinces. Before the data collection studies, necessary data sets to be needed were determined to create resource usage profiles in textile sub-sectors. Then, data collection forms to be applied at the facilities were prepared. In general, prepared data collection forms contained the main activity area of the facility, water consumption, auxiliary chemical consumption, dyestuff consumption, electricity consumption, steam consumption, and production amount data.

In the selection of the facilities, criteria such as field of activity, process differences, production capacity, and location were taken into consideration. Thus, it was aimed to represent the general distribution of textile sub-sectors in Turkey as much as possible. Within the scope of this study, data collection studies were carried out in a total of 150 facilities. However, due to data quality and project agreement issues, data from 44 facilities was used in this paper. These facilities were in the sub-sectors of cotton woven fabric (41%), wool woven fabric (9%), synthetic woven fabric (7%), cotton knitted fabric (32%), synthetic knitted fabric (7%), and non-woven fabric (4%).

Data collection forms were applied to the textile industries located in the determined regions. Consumption (water, auxiliary chemicals, dyestuff, electricity, and steam) and production data were collected from both main production and auxiliary processes of the facilities for the last three years (2018–2020). Average annual production, average water consumption, average auxiliary chemical consumption, average dyestuff consumption, average electricity consumption, and average steam consumption were calculated facility. Using the average annual water consumption and average annual production data, the average specific water consumption was calculated for each textile sub-sector (Eq. 1). Average specific water consumptions were evaluated under six range groups: < 50, 50–100, 100–150, 150–200, 200–250, and > 250 L/kg product.

1 $$\mathrm{Average\; specific\; water\; consumption }\;({\text{L}}/\mathrm{kg \;product})=\mathrm{Average\; annual \;water\; consumption}\;({\text{L}}/{\text{year}})/\mathrm{Average\; annual\; production\; value }\;({\text{kg}}/{\text{year}})$$

Average specific chemical (auxiliary chemical and dyestuff) consumption was calculated using Eq. 2. Average specific auxiliary chemical consumptions were evaluated under six range groups: < 100, 100–200, 200–300, 300–400, 400–500, and > 500 g/kg product. Average specific dyestuff consumptions were also evaluated under 6 range groups: < 10, 10–20, 20–30, 30–40, 40–50, and > 50 g/kg product. The average specific energy consumption (electricity and steam) for each facility was calculated using Eqs. 3 and 4. In addition, the evaluated facilities were classified based on sub-sectors according to their field of activity. Average specific consumption values for the sub-sectors were found by calculating the averages and standard deviations of the average specific consumption calculated for each facility. Standard deviation was also calculated within a sub-sector containing multiple facilities using Eq. 5.

2 $$\mathrm{Average}\;\mathrm{specific}\;\mathrm{chemical}\;\mathrm{consumption}\;(\text{g}/\mathrm{kg}\;\mathrm{product})=\mathrm{Average}\;\mathrm{annual}\;\mathrm{chemical}\;\mathrm{consumption}\;(\text{kg}/\text{year})/\mathrm{Average}\;\mathrm{annual}\;\mathrm{production}\;\mathrm{value}\;(\text{kg}/\text{year})\times1000$$

Average specific electricity consumptions were evaluated under five range groups: < 1, 1–2, 2–3, 3–4, and > 4 kWh/kg product.

3 $$\mathrm{Average}\;\mathrm{specific}\;\mathrm{electricity}\;\mathrm{consumption}\;(\text{kWh}/\mathrm{kg}\;\mathrm{product})=\mathrm{Average}\;\mathrm{annual}\;\mathrm{electricity}\;\mathrm{consumption}\;(\text{kWh}/\text{year})/\mathrm{Average}\;\mathrm{annual}\;\mathrm{production}\;\mathrm{value}\;(\text{kg}/\text{year})$$

Average specific steam consumptions were evaluated under five range groups: < 10, 10–20, 20–30, 30–40, and > 40 kg steam/kg product.

4 $$\mathrm{Average}\;\mathrm{specific}\;\mathrm{steam}\;\mathrm{consumption}\;(\mathrm{kg}\;\mathrm{steam}/\mathrm{kg}\;\mathrm{product})=\mathrm{Average}\;\mathrm{annual}\;\mathrm{steam}\;\mathrm{consumption}\;(\mathrm{kg}\;\mathrm{steam}/\text{year})/\mathrm{Average}\;\mathrm{annual}\;\mathrm{production}\;\mathrm{value}\;(\text{kg}/\text{year})$$

5 $$\sigma =\sqrt{\frac{1}{n}\sum_{i=1}^{n}{\left({x}_{i}-\overline{x }\right)}^{2}}$$

σ: Standard deviation

*n*: The sample size

*x*_*i*_: Variant values

$\overline{x }$: Average value (Wan et al. 2014; Martinez and Bartholomew 2017).

Specific water consumption, specific auxiliary chemical consumption, specific dyestuff consumption, specific electricity consumption, and specific steam consumption values reported in the TXT BREF and the literature based on sub-sectors were investigated. Average potential reduction ratios were calculated by comparing the average specific consumptions calculated for each facility with the minimum value reported for similar textile mills in the TXT BREF and the literature (Eq. 6). The average reduction ratio in each sub-sector was calculated using the average and standard deviation values of the average reduction ratios found for all facilities evaluated in the relevant sub-sector. Accordingly, sub-sector profiles including specific resource uses (water, chemicals and energy) and potential reduction ratios of the Turkish textile sector were revealed. Potential water, auxiliary chemical, dyestuff, electricity, and steam reductions were evaluated under four range groups: 0–25, 25–50, 50–75, and 75–100%.

6 $$\mathrm{Potential}\;\mathrm{reduction}\;\mathrm{ratio}\left(\%\right)=\frac{\left(\mathrm{Average}\;\mathrm{specific}\;\mathrm{value}\;\mathrm{of}\;\mathrm{the}\;\mathrm{facility}\right)-(\mathrm{Reported}\;\mathrm{minimum}\;\mathrm{specific}\;\mathrm{value})}{\mathrm{Average}\;\mathrm{specific}\;\mathrm{value}\;\mathrm{of}\;\mathrm{the}\;\mathrm{facility}}\times100$$

In all figures given in this paper, the percentage value shown in each range group indicates the percentage of the number of facilities which falls in this range group.

## Results

Specific water, chemical, and energy consumptions of the textile sub-sectors in Turkey were determined, and potential reductions were calculated by comparing them with the data of TXT BREF and similar global textile industries. Sub-sector profiles were revealed showing resource use and reduction potentials. The results and discussions are presented under the following sub-headings.

### Specific water consumption and potential reduction ratios

**Fig. 1:**
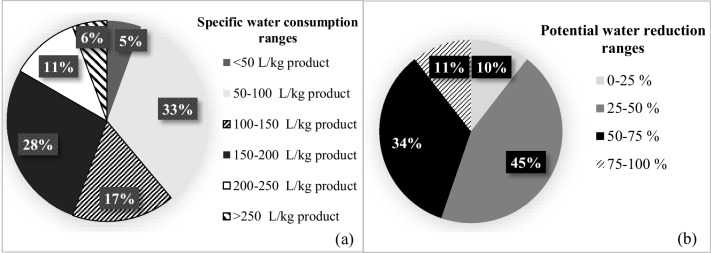
Distribution of specific water consumptions in the cotton woven fabric dyeing-finishing sub-sector (**a**) and distribution of potential water reduction ranges for the whole sector (**b**)

A total of 25 textile facility engaged in woven fabric dyeing-finishing sub-sector was analyzed. Among these facilities, 72% were cotton, 16% woolen, and 12% synthetic woven fabric producers. The average specific water consumption was found to be 134 ± 58 L/kg product for cotton woven fabric dyeing-finishing. The distribution of specific water consumptions in the investigated sub-sectors are presented in Fig. 1.

Average specific water consumptions for each sub-sector, reference specific water consumption values reported in TXT BREF documents and literature, and calculated potential reduction ratios are given in Table 1. The average water reduction potential in the woven fabric dyeing-finishing sub-sector was found to be 63 ± 15%. Moreover, when evaluated with respect to percent reduction ranges, 19% of the facilities in this sub-sector were in 25–50% range, 62% were in 50–75% range, and 19% were in 75–100% range.

**Table 1:** Specific water consumptions and reduction potentials

Textile sub-sectors		Average specific water consumption^1^ (L/kg product)	Reference value^2^ (L/kg product) (min.-max.)	Potential reduction ratio (%)
Woven fabric dyeing and finishing	Cotton	134 ± 58	45–200	63 ± 15
	Wool	345 ± 262	90–400	63 ± 20
	Synthetic	73 ± 15	100–248	-^3^
Knitted fabric dyeing and finishing	Cotton	90 ± 16	60–136	31 ± 12
	Synthetic	61 ± 9	35–229	42 ± 9
Non-woven fabric production	Synthetic	55 ± 50	0.45–91^4^	58

^1^It contains the data of 44 facilities in total. 41% of the facilities are in cotton woven fabric, 9% wool woven fabric, 7% synthetic woven fabric, 32% cotton knitted fabric, 7% synthetic knitted fabric, and 4% non-woven fabric sub-sectors

^2^EC (2003); Fuchs et al. (2004); Alkaya and Demirer (2014); Muthu (2019); Ozturk and Cinperi (2018); Hossain and Khan (2020); Zhu (2022); Haque et al. (2021); Okafor et al. (2021); Chakraborty and Ahmad (2022);Roth et al. (2023)

^3^Average specific water consumption could not be calculated as it was below the reported reference values

^4^Karthik and Gopalakrishnan (2014);Ozturk (2022)

The average specific water consumption was calculated as 345 ± 262 L/kg product in the wool woven fabric dyeing-finishing sub-sector. Low number of facilities investigated in this sub-sector and the heterogeneous nature of such facilities resulted in a high standard deviation value. It was calculated that 50% of the evaluated facilities in the wool woven fabric dyeing-finishing sub-sector have an average specific water consumption between 150 and 250 L/kg product and 50% of them have an average specific water consumption over 250 L/kg product. Once the average specific water consumption of the facilities evaluated in this sub-sector was compared with similar textile facility data in the TXT BREF documents and the literature, it was found that there was an average of 63 ± 20% water consumption reduction potential (Table 1). With respect to percent reduction ranges, following trend was found; 50% of the facilities evaluated in this sub-sector: 25–50%, 25% of the facilities: 50–75% and 25% of the facilities: 75-–100%.

The average specific water consumption value for the synthetic woven fabric dyeing-finishing sub-sector was calculated as 73 ± 15 L/kg product. It was found that all of the facilities evaluated in this sub-sector have an average specific water consumption value between 50 and 100 L/kg product. It was found that the calculated average for this sub-sector was below the minimum values reported for comparable textile facilities. Moreover, it was found that 25% of the facilities have the potential to reduce their water consumption by 25–50%, 55% by 50–75%, and 20% by 75–100%.

A total of 17 textile facilities engaged in the dyeing-finishing of knitted fabrics was included in this study. Within these facilities, 82% was manufacturers of cotton fabric and the remaining 18% was manufacturers of synthetic knitted fabric. The average specific water consumption for the cotton knitted fabric dyeing-finishing sub-sector was calculated as 90 ± 16 L/kg product. It was found that 71% of the facilities in this sub-sector have average specific water consumption values in the 50–100-L/kg product range, and 29% have average specific water consumption values in the 100–150-L/kg product range. After comparing the average specific water consumption of the facilities in this sub-sector with similar data from the TXT BREF documents and literature, it was found that there is a potential for an average reduction of 31 ± 12% (Table 1). On the other hand, it was found that 29% of the facilities in this sub-sector could reduce their water consumption by less than 25%, while 71% had the potential to reduce their water consumption by 25–50%.

The average specific water consumption value in the synthetic knit fabric dyeing-finishing sub-sector was calculated as 61 ± 9 L/kg product. It was found that all facilities performing synthetic knitted fabric dyeing-finishing had a specific water consumption value in the range of 50–100 L/kg product. It was found that the synthetic knitted fabric dyeing-finishing sub-sector had an average reduction potential of 42 ± 9% (Table 1). Moreover, it was found that 33% of the facilities operating in the synthetic knitted fabric dyeing-finishing sub-sector had a water reduction potential range of 50–75% and the remaining 67% had the range of 25–50%. For the overall knitted fabric dyeing-finishing sub-sector, it was found that 23% of the evaluated facilities had a water reduction potential of < 25%, 71% had a water reduction potential of 25–50%, and 6% had a water reduction potential of 50–75%.

In the non-woven fabric production sub-sector, the average specific water consumption value was found to be 55 ± 50 L/kg product (Table 1). It was found that half of the facilities evaluated in the non-woven fabric dyeing-finishing sub-sector had specific water consumptions of < 50 L/kg product and the other half was in the 100–150-L/kg product range. When such values were compared with those in the TXT BREF documents and literature, the potential reduction ratio was found to be 58% on average. Moreover, it was calculated that the water consumption reduction potential of all facilities evaluated in this sub-sector varied between 50 and 75%. The distributions of potential water reductions in all textile sub-sectors are presented in Fig. 1.

### Specific auxiliary chemical-dyestuff consumption and potential reduction ratios

**Fig. 2:**
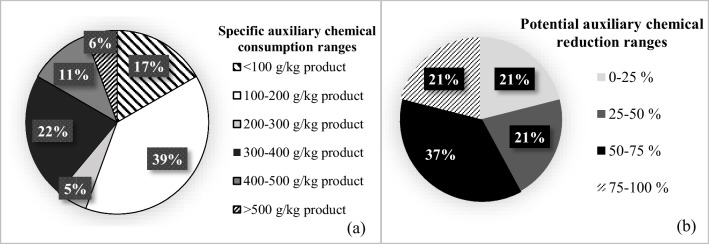
Distribution of specific auxiliary chemical consumptions in cotton woven fabric dyeing-finishing sub sector (**a**) distribution of potential auxiliary chemical reduction ranges for the whole sector (**b**)

In the cotton woven fabric dyeing-finishing sub-sector, the average specific auxiliary chemical consumption was estimated as 231 ± 165 g/kg product. Average specific auxiliary chemical consumptions, average specific dye consumptions, reference values, and potential reduction ratios for each sub-sector are presented in Table 2. The distribution of specific auxiliary chemical consumptions of the evaluated facilities is shown in Fig. 2. Although the average specific auxiliary chemical use in the cotton woven fabric sub-sector remains below the reported reference values, the average specific auxiliary chemical consumption in approximately 39% of the facilities evaluated in this sub-sector is above 300 g/kg product value (Fig. 2). The potential auxiliary chemical reduction ratio was calculated as 18 ± 15% for this sub-sector. In addition, it was calculated that 75% of the evaluated facilities have a reduction potential of < 25% and remaining 25% of the facilities have 25–50% reduction potentials. The distributions of potential auxiliary chemical reduction ratios in all textile sub-sectors are also presented in Fig. 2.

**Table 2:** Specific chemical consumptions and reduction potentials

Textile sub-sectors		Average specific auxiliary chemical consumption^1^ (g/kg product)	Average specific dyestuff consumption (g/kg product)	Reference value for auxiliary chemical^2^ (g/kg product) (min.-max.)	Reference value for dyestuff^2^ (g/kg product) (min.-max.)	Potential auxiliary chemical reduction ratio (%)	Potential dyestuff reduction ratio (%)
Woven fabric dyeing and finishing	Cotton	231 ± 165	23 ± 14	380–450	10–50	18 ± 15	61 ± 17
	Wool	221 ± 171	21 ± 16	225–255	10–30	33 ± 27	49 ± 36
	Synthetic	397 ± 237	20 ± 10	130–220	10–50	76 ± 10	60 ± 10
Knitted fabric dyeing and finishing	Cotton	367 ± 232	30 ± 13	270–670	18	47 ± 14	37 ± 20
	Synthetic	376 ± 96	30 ± 7	95–430	15–50	73 ± 8	47 ± 14
Non-woven fabric production	Synthetic	244 ± 40	18 ± 1	-^3^	-	-	-

^1^It contains the data of 44 facilities in total. 41% of the facilities are in cotton woven fabric, 9% wool woven fabric, 7% synthetic woven fabric, 32% cotton knitted fabric, 7% synthetic knitted fabric, and 4% non-woven fabric sub-sectors

^2^European Commission (EC) (2003); Kalliala and Talvenmaa (2000); Ozturk et al. (2016a); Ozturk et al. (2016b); Ozturk et al. (2020b); Simsek et al. (2022);Roth et al. (2023)

^3^Data not available

**Fig. 3:**
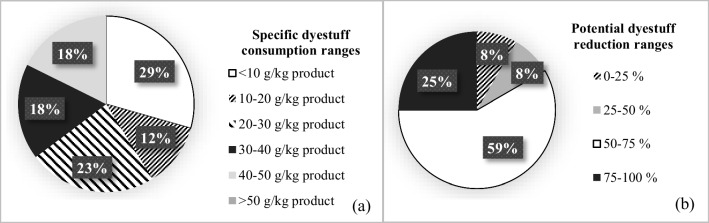
Distribution of specific dyestuff consumptions (**a**) and distribution of potential dyestuff reduction ratios in the cotton woven fabric dyeing-finishing sub-sector (**b**)

**Fig. 4:**
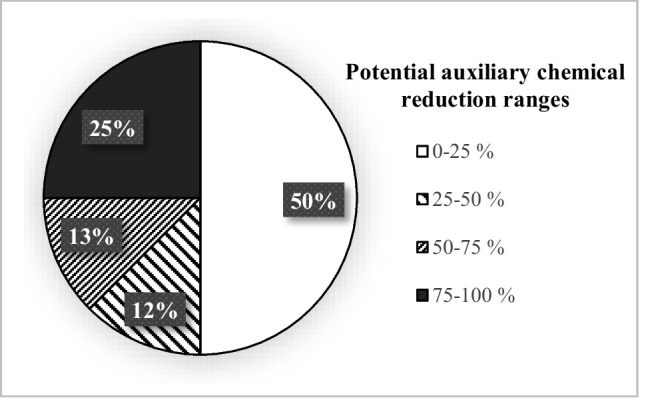
Distribution of total potential auxiliary chemical reduction ratios in across the woven fabric dyeing-finishing sub-sector

**Fig. 5:**
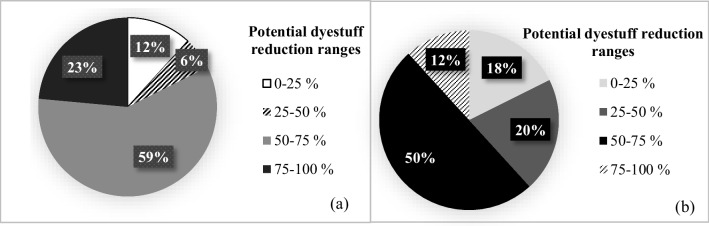
Distribution of total potential dyestuff reduction ratios across the woven fabric dyeing-finishing sub-sector (**a**) and distribution of potential dyestuff reduction ranges for the whole sector (**b**)

The average specific dyestuff consumption in the cotton woven fabric sub-sector was found as 23 ± 14 g/kg product (Table 2). The distribution of specific dyestuff consumptions of the cotton woven fabric dyeing-finishing sub-sector is given in Fig. 3. It was found that there is an average of 61 ± 17% reduction potential when average specific dyestuff consumption values were compared with the reported minimum specific values in the TXT BREF and the literature. The distribution of total potential auxiliary chemical reduction ratios for the entire woven fabric dyeing-finishing sub-sector is given in Fig. 4. Potential dyestuff reduction ratios of the cotton woven fabric dyeing-finishing sub-sector are presented in Fig. 5. The distributions of potential dyestuff reduction ratios in all textile sub-sectors are presented in Fig. 5.

The average specific auxiliary chemical consumption in the wool woven fabric dyeing-finishing sub-sector was calculated as 221 ± 171 g/kg product (Table 2). Distribution of the facilities based on consumption ranges was as following; 25% of the facilities: < 100, 25%: 100–200, 25%: 200–300, and 25%: 400–500 g/kg product. In addition, it was found that there is an average reduction potential of 27 ± 15% when the average specific auxiliary chemical consumption value calculated for this sub-sector is compared with the reported minimum values. Half of the facilities evaluated in this sub-sector were found to have an auxiliary chemical reduction potential of < 25% and the other half have 50–75% potential.

The average specific dyestuff consumption in the wool woven fabric sub-sector was calculated as 21 ± 16 g/kg product. The distribution was as follows; 25% of the facilities evaluated in this sub-sector: < 10, 25%: 10–20, 25%: 20–30, and remaining 25%: 40–50 g/kg product. It was calculated that there is an average of 49 ± 36% dyestuff reduction potential in the woolen woven fabric dyeing-finishing sub-sector (Table 2). Moreover, it was found that 34% of the facilities evaluated in this sub-sector had a dyestuff reduction potential of < 25%. Data for the remaining was 33% of facilities: 50–75% and 33% of facilities: 75–100%.

The average specific auxiliary chemical consumption in the synthetic woven fabric dyeing-finishing sub-sector was found to be very variable with an average of 397 ± 237 g/kg product, while the average specific dyestuff consumption in the same sub-sector was 20 ± 10 g/kg product. It was found that 33% of the facilities evaluated in the synthetic woven fabric dyeing-finishing sub-sector had specific auxiliary chemical consumption in the 100–200-g/kg product range. The remaining 67% of the facilities had values over 500 g/kg product. It was found that 34% of the facilities had specific dyestuff consumptions of less than 10 g/kg product. Meanwhile, 33% of the facilities exhibited dyestuff consumption range of 20–30 g/kg product, while the remaining 33% was in 30–40 g/kg product range. When the specific auxiliary chemical and specific dyestuff consumptions calculated for this sub-sector are compared with the minimum values reported, reduction potentials of 76 ± 1% and 60 ± 10% were calculated, respectively (Table 2). The potential reductions in the consumption of auxiliary chemicals and dyestuff for this sub-sector were found to be 75–100% and 50–75%, respectively. The distribution of potential auxiliary chemical and dyestuff reduction ratios for the whole woven fabric dyeing-finishing sub-sector is given in Figs. 4 and 5.

**Fig. 6:**
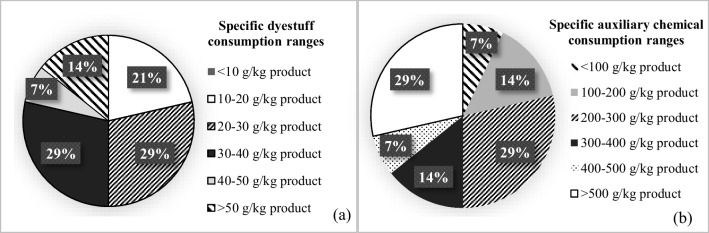
Distribution of specific dyestuff consumptions (**a**) and distribution of specific auxiliary chemical consumptions in knitted fabric dyeing-finishing sub-sector (**b**)

Average specific auxiliary chemical and average specific dyestuff consumption were found to be 367 ± 232 g/kg product and 30 ± 13 g/kg product in the cotton knitted fabric sub-sector, respectively. The distributions are shown in Fig. 6. There is an average of 47 ± 10% and 37 ± 20% reduction potentials in auxiliary chemical and dyestuff consumptions in this sub-sector, respectively (Table 2). Auxiliary chemical reduction ranges were 25–50% and 50–75% for 43% and 57% of the studied facilities in this sub-sector, respectively. For dyestuff, 25% of the facilities exhibited potential reductions less than 25%. The remaining 33% and 42% of the studied facilities were in 25–50% and 50–75% reduction ranges, respectively.

The average specific auxiliary chemical consumption was calculated as 376 ± 96 g/kg product in the synthetic knitted fabric sub-sector. The average specific dyestuff consumption for the same sub-sector was 30 ± 7 g/kg product. It was observed that in 33% of the facilities assessed in this sub-sector, the specific auxiliary chemical consumption range was 200–300 g/kg product, while in 67% of them, the range was 400–500 g/kg product. Similarly, in 33% and 67% of the same facilities, specific dyestuff consumption ranges were 20–30 and 30–40 g/kg product, respectively. Potential reductions in auxiliary chemical and dyestuff consumptions were calculated as 73 ± 8% and 47 ± 14%, respectively (Table 2). It was determined that 33% of the synthetic knitted fabric dyeing-finishing enterprises had 50–75% reduction potential of auxiliary chemicals and 25–50% reduction potential of dyestuffs. For the remaining 67% facilities, auxiliary chemical and dyestuff reduction potentials were 75–100 and 50–75%, respectively.

In the non-woven fabric sub-sector, the average specific auxiliary chemical consumption was found to be 244 ± 40 g/kg product and the average specific dyestuff consumption was 18 ± 1 g/kg product (Table 2). It was found that 50% of non-woven fabric dyeing-finishing facilities consume less than 100 g/kg of auxiliary chemicals, while the other 50% consume 400–500 g/kg. In all the facilities of this sub-sector, the specific dyestuff consumption range was 10–20 g/kg product. The TXT BREF does not contain data on the consumption of specific auxiliary chemicals and dyestuffs for this sub-sector. Thus, potential reduction ratios could not be calculated for this sector.

In the knitted fabric dyeing-finishing sub-sector, it was found that 30% of the facilities exhibited auxiliary chemical reduction potential of less than 25%. Such ranges were 50–75 and 75–100% for the remaining 50 and 20% of the facilities, respectively. In terms of dyestuff, 20% of the facilities showed less than 25% reduction potential. The remaining 33 and 47% of the studied facilities in this sub-sector had 25–50 and 50–75% ranges, respectively.

### Specific energy consumption and potential reduction ratios

#### Electricity

**Fig. 7:**
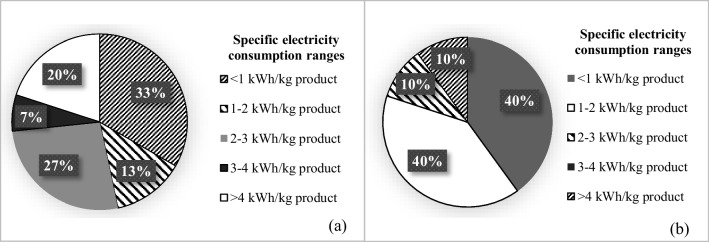
Distribution of specific electricity consumptions in cotton woven fabric dyeing-finishing sub-sector (**a**) and cotton knitted fabric dyeing-finishing sub-sector (**b**)

Average specific electricity consumptions, reported reference values, and calculated average potential electricity reduction ratios are presented in Table 3. The average specific electricity consumption in the cotton woven fabric dyeing-finishing sub-sector was calculated as 2.8 ± 2.6 kWh/kg product (Table 3). The distribution of specific electricity consumption values of the facilities evaluated in this sub-sector is given in Fig. 7. When compared with the reported minimum values, the average specific electricity consumption calculated for this sub-sector can be reduced by 66 ± 22%. It was found that 34% of the evaluated facilities in this sub-sector have the potential to reduce their electricity consumption by 25–50%, 13% by 50–75%, and 53% by 75–100%.

**Table 3:** Specific electricity consumptions and reduction potentials

Textile sub-sectors		Average specific electricity consumption^1^ (kWh/kg product)	Reference value for electricity^2^ (kWh/kg product) (min.-max.)	Potential electricity reduction ratio (%)
Woven fabric dyeing and finishing	Cotton	2.8 ± 2.6	0.5–1.5	66 ± 22
	Wool	7 ± 5.3	0.5–0.8	71 ± 17
	Synthetic	1.65 ± 0.24	0.5–1.5	69 ± 4.5
Knitted fabric dyeing and finishing	Cotton	1.58 ± 1.3	1–3	39 ± 20
	Synthetic	1.2 ± 0.7	1.5–6	Under the reference value
Non-woven fabric production	Synthetic	3.3 ± 0.7	0.9–6.5	50 ± 25

^1^It contains the data of 44 facilities in total. 41% of the facilities are in cotton woven fabric, 9% wool woven fabric, 7% synthetic woven fabric, 32% cotton knitted fabric, 7% synthetic knitted fabric, and 4% non-woven fabric sub-sectors

^2^European Commission (EC) (2003); Visvanathan et al. (2000); IEE (2006); Hasanbeigi (2010); Velden et al. (2014); Ozturk et al. (2020a); Simsek et al. (2022); Roth et al. (2023)

The average specific electricity consumption was found to be 7 ± 5.3 kWh/kg product in the wool woven fabric production sub-sector (Table 3). It was found that half of the facilities in this sub-sector had specific electricity consumption range of 3–4 kWh/kg product, while the other half had specific electricity consumption above 4 kWh/kg product. The potential electricity reduction ratio for this textile sub-sector was found to be 71 ± 17%. It was found that 50% of the facilities in this sub-sector had the potential to reduce their electricity consumption by 50–75%. The remaining 50% of the facilities could reduce it by 75–100%.

The average specific electricity consumption in textile facilities producing synthetic woven fabrics was calculated as 1.65 ± 0.2 kWh/kg product. Average specific electricity consumption values of all facilities evaluated in this sub-sector were between 1 and 2 kWh/kg product. It was found that there is an electricity reduction potential of 69 ± 4.5% in this sub-sector when the calculated average specific electricity consumption is compared with the reported minimum reference values. It was calculated that 23% of the facilities evaluated in this category had the potential to reduce their electricity consumption by 25–50%. The remaining 32 and 45% of the facilities exhibited 50–75 and 75–100% reduction potentials, respectively.

The average specific electricity consumption was found as 1.58 ± 1.3 kWh/kg product for the cotton knitted fabric sub-sector (Table 3). The distributions are given in Fig. 7. It was found that there is an average of 39 ± 30% electricity reduction potential in this sub-sector. It was determined that among the facilities assessed in this sub-sector, 20% had the potential to reduce their electricity consumption by less than 25%, while 40% had potential in the range of 25–50%. The remaining 20 and 20% of the facilities exhibited potentials in 50–75 and 75–100% ranges, respectively.

**Fig. 8:**
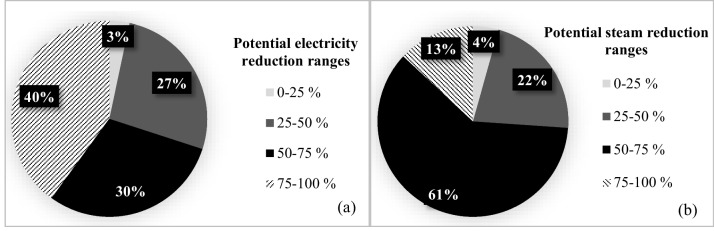
Distribution of potential electricity reduction ranges (**a**) and distribution of potential steam reduction ranges for the whole sector (**b**)

In the synthetic knitted fabric sub-sector, the specific average electricity consumption was calculated as 1.2 ± 0.7 kWh/kg product (Table 3). It was found that 34% of the evaluated facilities in this sub-sector had specific electricity consumption of < 1, 33% had 1–2, and the remaining 33% had 2–3 kWh/kg product. It was concluded that the average specific electricity consumption in the synthetic knitted fabric sector remains under the reported reference values. When potential electricity consumption reduction ratios were evaluated for the entire knitted fabric dyeing-finishing sub-sector, it was found that 16% of the facilities had potentials below 25%. The remaining 50, 17, and 17% of the facilities were in the 25–50, 50–75 and 75–100% reduction ranges, respectively. The distributions of potential electricity reduction ratios in all the studied textile sub-sectors are presented in Fig. 8.

In the non-woven fabric sub-sector, the average specific electricity consumption was found to be 3.3 ± 0.7 kWh/kg product. In about half of the facilities evaluated in this sub-sector, the specific electricity consumption was between 2 and 3 kWh/kg product. In the other half, such values were between 3 and 4 kWh/kg product. When the average specific electricity consumption value calculated for the non-woven sub-sector is compared with the reported minimum values, it was found that there is a reduction potential of 50 ± 25% (Table 3). It was found that about 50% of the facilities in this sub-sector had electricity consumption reduction potential of 50–75%. The remaining half was in 75–100% range.

#### Thermal energy

Table 4 shows the calculated average specific steam consumptions, reported reference values and potential reduction ratios. The consumption was found to be 13.8 ± 4.7 kg steam/kg product in the cotton woven fabric dyeing-finishing sub-sector. It was found that 31% of the facilities in this sub-sector had specific steam consumptions below 10 kg steam/kg product. The remaining 61 and 8% of the facilities fell in the 10–20- and 20–30-kg steam/kg product ranges, respectively. Calculated average specific steam consumption was compared with the reported minimum values and 50 ± 16% steam reduction potential on average was found in this sub-sector (Table 4). It was found that 8% of the evaluated facilities in this sub-sector had a steam reduction potential of less than 25%. The remaining 25 and 67% of the facilities were in the 25–50 and 50–75% range, respectively.

**Table 4:** Specific steam consumptions and reduction potentials

Textile sub-sectors		Average specific steam consumption^1^ (kg steam/kg product)	Reference value for steam^2^ (kg steam/kg product) (min.-max.)	Potential steam reduction ratio (%)
Woven fabric dyeing and finishing	Cotton	13.8 ± 4.7	4–14	50 ± 16
	Wool	20 ± 11	4–14	75 ± 13
	Synthetic	6.8 ± 1	4–14	42
Knitted fabric dyeing and finishing	Cotton	11.3 ± 6	4–14	63 ± 20
	Synthetic	8.5 ± 4	4–14	64
Non-woven fabric production	Synthetic	-^3^	-	-

^1^It contains the data of 44 facilities in total. 41% of the facilities are in cotton woven fabric, 9% wool woven fabric, 7% synthetic woven fabric, 32% cotton knitted fabric, 7% synthetic knitted fabric, and 4% non-woven fabric sub-sectors

^2^European Commission (EC) (2003); Asian Regional Research Programme in Energy Environment and Climate (ARRPEEC) (2003); Purohit (2007); Hasanbeigi (2010); Pakistan Cleaner Production Institute (PCPI) (2014); Roth et al. (2023)

^3^Data not available

The average specific steam consumption was found to be 2 0 ± 11 kg steam/kg product in the wool woven fabric sub-sector. It was found that 25% of the facilities assessed in this sub-sector had specific steam consumptions below 10 kg steam/kg product. The remaining 50 and 25% of the facilities were in 10–20 and 30–40 kg steam/kg product ranges, respectively. There is a 75 ± 13% steam reduction potential in this textile sub-sector, when benchmarked with respect to reported minimum specific steam consumption values (Table 4). It was found that 75% of the facilities in this sub-sector had the potential to reduce steam by 50–75%, while 25% had the potential to reduce steam by 75–100%.

The average specific steam consumption in the synthetic woven fabric sub-sector was calculated as 6.8 ± 1 kg steam/kg product. It was found that the specific steam consumption was less than 10 kg steam/kg product in all of the facilities examined in this sub-sector. There is an average of 42% steam reduction potential in this sub-sector (Table 4). The steam consumption reduction potential of all facilities examined in this sub-sector varied between 25 and 50%. For all the facilities in the woven fabric dyeing-finishing sub-sector, the following reduction potentials in steam were obtained; 6% of the facilities: < 25%, 23%: 25–50%, 65%: 50–75%, and 6%: 75–100%.

In the cotton knit fabric dyeing-finishing sub-sector, the average specific steam consumption was found to be 11.3 ± 6 kg steam/kg product. Among the evaluated facilities in this sub-sector, 75% of them had specific steam consumptions less than 10 kg steam/kg product. The remaining 12 and 13% of the facilities exhibited specific steam consumptions of 20–30 and 30–40 kg steam/kg product, respectively. The average potential steam reduction ratio in this sub-sector was found to be 63 ± 20% (Table 4). The following reduction potentials in steam were obtained; 20% of the facilities: 25–50%, 40%: 50–75%, and 40%: 75–100% (Fig. 8).

The average specific steam consumption was found as 8.5 ± 4 kg steam/kg product in the synthetic knitted fabric sub-sector. The specific steam consumption in all facilities examined in this sub-sector was below 10 kg steam/kg product. It was found that 64% steam reduction on average could be obtained in this sub-sector. The potential reduction in steam consumption was found to be between 50 and 75% in all facilities examined in this sub-sector. For the knitted fabric dyeing-finishing sub-sector, the following steam reduction potentials were found; 17% of the facilities: 25–50%, 50%: 50–75%, and 33%: 75–100%.

## Discussion

### Water consumptions

#### Cotton woven fabric dyeing-finishing sub-sector

The most important portion of the Turkish textile industry in terms of production capacity and number of facilities is the cotton woven fabric dyeing-finishing sub-sector. This sub-sector generally remains within the reference value ranges in terms of average specific water use, indicating that this sub-sector is in good shape in terms of water consumption when evaluated on a global scale. Approximately 55% of the facilities evaluated in this sub-sector have an average specific water consumption value less than 150 L/kg product (Fig. 1). The facilities with low specific water consumption are mostly small-scale and new dyehouses, which have practices to reduce water consumption and generally do not have a lot of product diversity (such as only pile fabric production). Facilities with high and very high average specific water consumption are mostly integrated facilities. These ones have long wet process chains (facilities with the mercerization process and printing unit), have wide product ranges, generally have old technology machinery (such as high liquor ratio), and do not have adequate water consumption reduction practices. Although the average specific water consumption in this sub-sector is within the reference value range, when the potential water reduction ratios are examined, it is found that there is a significant water reduction potential. In order to further reduce water consumption in the cotton woven fabric dyeing-finishing sub-sector, following measures are recommended: good management practices (such as monitoring specific water consumption on a process basis, establishment of a cleaner production system) (Yu et al. 2022), machine replacement or modifications (new technology machines with lower liquor ratio providing water efficiency), process optimization (reducing the number of after-wash baths, avoiding overflow washing), optimizing regeneration duration and frequency using hardness sensors in water softening systems, and reusing wastewater (with or without treatment).

#### Woolen woven fabric dyeing-finishing sub-sector

The woolen woven fabric sub-sector has an important capacity but a modest place in terms of facility number among all textile sub-sectors in Turkey. Facilities that dye and finish woolen woven fabrics in Turkey are generally integrated facilities and have high production capacities. In this study, approximately 80% of the facilities producing woolen woven fabrics in Turkey was represented. Although wool woven fabric dyeing-treatment has similarities with cotton fabric dyeing-treatment, it has important process and application differences. Specific water consumptions in facilities operating in this sub-sector are much higher than those of cotton fabric dyeing-finishing. The properties of the wool fiber and the greater number of wet process steps affect this. Although the average specific water consumption values of the facilities evaluated in this sub-sector remain within the reference value ranges, they are generally closer to the maximum reference value. This finding indicates a significant water consumption reduction potential in the woolen woven fabric sub-sector (Table 1). The water reduction measures listed above for the cotton woven fabric sub-sector are also valid for this sub-sector.

#### Synthetic woven fabric dyeing-finishing sub-sector

The average specific water consumption values of the synthetic woven fabric dyeing-finishing sub-sector are much less compared to the cotton and woolen fabric dyeing-finishing sub-sectors. There is limited data for reference values of this sub-sector in the TXT BREF Document, which makes the performance comparisons difficult. Overall, it can be stated that specific water consumption performances of the evaluated facilities in this sub-sector are relatively good. Woven fabric dyeing-finishing requires fewer fine process steps compared to other natural fabrics. Synthetic woven fabric finishing dyeing is usually processed in high-temperature (HT) dyeing machines. Pretreatment and finishing of synthetic fabrics require a shorter process chain than fabrics woven from natural fibers. Therefore, the average specific water consumption in synthetic fabric production is relatively much less. However, this does not mean that water consumption cannot be further reduced. The following measures can be applied: using machines with much lower liquor ratio, monitoring and control applications on a process basis, process optimization applications, and water optimization in auxiliary processes (e.g. optimizing regeneration frequencies and durations using online hardness sensors in water softening units, reuse of reverse osmosis (RO) concentrates). Furthermore, it is possible to reduce specific water consumption in this sub-sector and other textile sub-sectors by reusing segregated wastewater streams with or without treatment.

#### Cotton knitted fabric dyeing-finishing sub-sector

Although the cotton knit fabric dyeing-finishing sub-sector is in good condition in terms of average specific water consumption values, it also has a significant water reduction potential (Table 1). It was found that 71% of the facilities in this sub-sector have average specific water consumption in the range of 50–100 L/kg product with water reduction potential of 25–50%. This potential can be realized in this sub-sector by implementing water reduction practices previously expressed for the woven fabric dyeing-finishing sub-sector.

#### Synthetic knitted fabric dyeing-finishing sub-sector

The average specific water consumption values of the synthetic knitted fabric dyeing-finishing sub-sector remain within the reported reference value ranges and it can be said that this sub-sector is in good condition. Synthetic fabrics require fewer pre-treatment and finishing steps compared to fabrics made from natural fibers. Synthetic fabrics can generally be processed in a single dyeing machine, and pre-treatment and dyeing processes can be done in a single bath. Due to these technical features, water consumption is generally relatively less in synthetic fabric dyeing-finishing. However, further water reduction can be achieved in the facilities in this sub-sector with the practices recommended above for other sub-sectors. Another advantage of the facilities employing dyeing-finishing of synthetic fabrics is that the wastewater reuse opportunities are quite high since the wastewater pollution loads are relatively lower.

#### Non-woven fabric production

Non-woven fabric production is quite different from conventional textile dyeing-finishing in terms of both applied techniques and processes. In this sub-sector, non-woven fabric production is carried out according to spun bond, spun lace, and meltdown methods. In spun lace production, the bonding of the fibers is done with the help of water. However, new technology machines are generally used and almost all of the water is reused. Besides, cooling water is generally used to cool the fibers in non-woven production. Some facilities also have air-cooling applications. Due to the high product portfolio in this sub-sector, reported reference value ranges and calculated average specific water consumption values also vary widely, resulting in high standard deviation values (Table 1). In a significant part of this sub-sector, machines with new technology are used and their water consumption is quite low. A significant portion of the facilities in this sub-sector has zero liquid discharge. In this sub-sector, water optimization applications, especially in auxiliary processes (e.g., optimizing water softening systems, reusing RO concentrates and employing closed-loop cooling systems), can contribute to reducing water consumption. Our literature search indicated that very few cleaner production studies have been carried out in this sub-sector. This may be due to relatively less resource use and environmental impacts in this sub-sector. However, it should be noted for this sub-sector that there is a wide range of product diversity, which may affect resource use performances. Therefore, more studies and new data are needed for this sub-sector. We believe that the data presented in this paper will be very beneficial to non-woven fabric production.

#### Evaluation of sub-sectors

After a comprehensive assessment of all sub-sectors in terms of their potential to reduce water consumption, it was found that 10% of the evaluated facilities had a reduction potential of less than 25%. However, the reduction potentials of the remaining 45, 34, and 11% of the facilities were 25–50, 50–75, and 75–100%, respectively (Fig. 1). These results highlight the fact that there is still much work to do in all the textile sub-sectors in reducing water consumptions. Detailed technical water control measures for each sub-sector were listed in previous paragraphs. However, some further actions on macro-scale are also needed. First, water resources should be better managed and water pricing should be based on each river basin employing total cost (resource and environmental costs) approach. Second, monitoring of water resources should be improved both in terms of quality and quantity. Third, sectoral water allocations should be regulated based on new criteria such as total cost approach and cleaner production. Fourth, for improved inspections and implementations, Turkish IPPC regulation should be finalized. Lastly, water efficiency should be promoted. The Turkish textile industry generally has an export-oriented production structure consisting of sub-contracted dyehouses. Global customers and international environmental standards (such as Green Deal) encourage and even force facilities to reduce their water footprints. Moreover, the pressures on the quantity of water resources such as global climate change and increased groundwater withdrawals also enforce the facilities to use water efficiently.

### Auxiliary chemical and dyestuff consumptions

#### Cotton woven fabric dyeing-finishing sub-sector

Cotton woven fabric sub-sector was found to be in moderate to good condition in terms of specific auxiliary chemical consumptions, when compared to the reference values. However, approximately 17% of the facilities in this sub-sector had specific auxiliary chemical consumptions above 400 g/kg product (Fig. 2), indicating a significant auxiliary chemical reduction potential. This sub-sector was at a medium level in terms of average specific dyestuff consumptions and had a significant dyestuff reduction potential. Cotton woven fabrics are generally dyed with reactive dyestuffs and they require high amounts of salt. This increases the use of auxiliary chemicals. In addition, facilities with high consumption of specific auxiliary chemicals and dyestuffs are generally integrated facilities with large-scale printing units. Especially in printing dyeing, chemical consumption can be high since there is a significant amount of printing paste losses. In other dye houses, the lack of automatic systems for dye preparation and dosing (it is done manually) is a factor which increases chemical consumption.

#### Woolen woven fabric dyeing-finishing sub-sector

Woolen woven fabric sub-sector was found to be in moderate level in terms of specific auxiliary chemical and dyestuff consumptions, when compared to the reference values. Potential to further reduce these consumptions exists in this sub-sector. A few of the facilities evaluated in this sub-sector have automatic paint kitchens and dosing systems. Reductions can be achieved by disseminating practices such as monitoring specific chemical consumption on a process basis, optimizing frequently used recipes, establishing automatic chemical preparation kitchens and automatic dosing systems, and recovery of chemicals from wastewaters (e.g. caustic recovery from mercerization, sizing agent recovery from desizing, reuse of washing chemical solutions). Implementations including wastewater reuse and chemical recovery can make significant contributions to circular economy approach in this sub-sector (Hora et al. 2023).

#### Synthetic woven fabric dyeing-finishing sub-sector

Unexpectedly, average specific auxiliary chemical consumptions in the synthetic woven fabric dyeing-finishing sub-sector were found to be much higher than the reported reference values. This was most probably due to high amounts of salt used in the regeneration of cationic ion exchange resins in softening units. The average specific dyestuff consumption in this sub-sector was within the reference values. However, a significant amount of auxiliary chemical and dyestuff reduction potential was found in this sub-sector, employing the previously mentioned practices.

#### Cotton and synthetic knitted fabric dyeing-finishing sub-sectors

Average specific auxiliary chemical consumptions in the cotton and synthetic knitted fabric sub-sectors were within the reference values, exhibiting a moderate level of performance. However, further potential reductions in auxiliary chemical consumptions are available in both sub-sectors. It was found that both of these sub-sectors can reduce their dyestuff consumptions by almost 50%.

#### Non-woven fabric production

The non-woven fabric production sub-sector is one of the sectors with high product and production diversity. Although a net comparison could not be made due to limited reference values, chemical use efficiency was found to be generally moderate to good level in this sub-sector. That is mainly because new technology machines are used and there is no need for high chemical consumptions other than raw materials in production processes.

#### Evaluation of all sub-sectors

When auxiliary chemical reduction ratios were examined in all sub-sectors, approximately 79% of the evaluated facilities were found to have reduction potentials more than 25%. Additionally, 82% of the evaluated facilities were found to have dyestuff reduction potentials more than 25%. Implementing the reduction practices mentioned above, as well as establishing smart dyehouses (computer-controlled or artificial intelligence supported), can significantly reduce chemical consumptions. Increasing such technology transfer practices as much as possible in order to keep up with the needs of the age in the sector will make significant contributions to sustainable textile production (Craiut et al. 2022). On the other hand, it is necessary to better analyze the relationship between water and chemicals in sub-sectors. In textile dyeing and finishing processes, chemicals are used to prepare a solution bath according to specific recipes. Therefore, the higher the water usage, the higher the chemical usage must be. Thus, reducing water consumption in textile dyeing and finishing processes will also reduce the chemical amounts. An important part of total production costs is chemicals, thus, reducing their amounts will also reduce costs. However, it is not easy to identify inefficiencies and apply chemical reduction techniques in facilities. Detailed studies should be carried out. Facilities generally hesitate to employ these practices due to their concerns on possible product quality changes. On the other hand, in recent years, the type and concentration of chemicals in textile wastewaters are being regulated by national and international regulations (e.g., EU Water Framework Directive). Global customers also dictate chemical-based environmental standards. Overall, auxiliary chemical and dyestuff management in the textile sector is very challenging due to above-mentioned reasons.

### Energy consumptions

#### Cotton woven fabric dyeing-finishing sub-sector

In the cotton woven fabric dyeing-finishing sub-sector, the average specific electricity consumption was above the reference values and there seems to be a significant electricity consumption reduction potential. It was found that 73% of the facilities had consumptions less than 3 kWh/kg product (Fig. 7). Average specific electricity consumption was very high, especially in integrated facilities (those engaged in spinning, weaving, dyeing, and finishing). In this sub-sector, electricity consumption can be reduced to a high extent by using more efficient electric motors, using speed and frequency drivers, optimizing compressed air and humidification systems, making lighting systems more efficient, and using renewable energy sources. An average 1.85 kWh/kg product reduction potential was found for electricity in the cotton woven fabric dyeing-finishing sub-sector. Although the average specific steam consumption values in this sub-sector were within the reference ranges, they were closer to the maximum reference values. The specific steam consumptions were generally high in integrated plants. Steam reductions can be achieved by optimizing steam boilers, insulating steam distribution systems and hot surfaces, monitoring specific energy (electricity and steam) consumption on a process basis, and heat recovery practices from waste gases and wastewaters. An average 6.9 kg steam reduction potential was found in steam per mass product in this sub-sector. Some of these practices already existed in most of the examined facilities. However, they were not adequate. Steam and heat losses were quite high especially in old facilities.

#### Woolen and synthetic woven fabric dyeing-finishing sub-sectors

The woolen woven fabric dyeing-finishing sub-sector was found to be problematic in terms of electricity and steam consumptions. The specific consumptions were above the reference values. The practices mentioned above for the cotton woven fabric dyeing-finishing sub-sector are also valid for this sub-sector. Implementation of these practices may provide significant energy reductions. Average 4.97 kWh/kg product and average 15 kg steam/kg product reduction potentials were found for electricity and steam in the woolen woven fabric dyeing-finishing sub-sector. Average specific electricity and steam consumptions in the synthetic woven fabric dyeing-finishing sub-sector remained within the reference values. However, further energy reduction potentials existed. An average 1.14 kWh/kg product reduction potential was found for electricity in the synthetic woven fabric dyeing-finishing sub-sector. The average steam reduction potential was found to be 2.86 kg steam/kg product.

#### Cotton and synthetic knitted fabric dyeing-finishing sub-sectors

Average specific electricity and steam consumptions in the cotton knitted fabric sub-sector were within the reference values. It was found that 80% of the evaluated facilities in this sub-sector had less than 2 kWh/kg product value (Fig. 7). The specific electricity consumption was higher especially in integrated facilities. An average 0.62 kWh/kg product reduction potential was found for electricity in the cotton knitted fabric dyeing-finishing sub-sector. The average steam reduction potential was found to be 7.1 kg steam/kg product in this sub-sector. In the synthetic knitted fabric dyeing-finishing sub-sector, the average specific electricity consumptions remained below the reported minimum reference value. Steam consumptions were within the reference values, indicating that this sub-sector is in good condition in terms of electricity and steam consumption. However, by the implementation of above measures, further reductions can be achieved. The average steam reduction potential was found to be 5.4 kg steam/kg product.

#### Non-woven fabric production

The non-woven fabric production sub-sector has a very heterogeneous structure due to product and process diversity. One of the most important process components in non-woven production is electricity. Extruder melting of synthetic chips (polypropylenes) is the major electricity consuming production process. Therefore, the specific electricity consumption is generally higher than other textile sub-sectors. In this subsector, the average electricity reduction potential was found to be 1.65 kWh/kg product. There is no steam consumption in non-woven fabric production.

#### Evaluation of sub-sectors

When the potentials for electricity and steam consumption reductions are examined for all the sub-sectors, significant levels of reduction potentials were found (Fig. 8). Energy constitutes one of the most important inputs in all textile sectors. It is also a major cost component. The economic saving to be achieved by reducing energy consumption is an important driving force for facilities to make energy efficiency investments. The environmental standards of global customers also encourage facilities to invest in energy efficiency practices. In addition, national and international regulations (e.g., The European Green Deal, Carbon Neutral Standards, Carbon Border Adjustment, and Environmental Product Declaration) also require energy efficiency and carbon footprint reduction.

As can be experienced in many on-site industrial studies, we also encountered some limitations and difficulties during our study, resulting in some weak points. One such challenge was the lack of adequate process-based water, energy and chemical data in some facilities. Some facilities also hesitated to share their data. While some sub-sectors were examined in greater depth, some others (i.e., non-woven fabric production) could not be evaluated in such detail due to limited number of facilities and inadequate data. Time and budget constraints of the project also limited the extension of the work. For future similar studies, we recommend the addition of other parameters such as specific wastewater amounts per mass of product, wastewater chemical loads, waste gas emissions, and solid wastes. Overall, this study drew a comprehensive picture of various textile sub-sectors in terms of their resource use and cleaner production performance. The potentials of reductions in water, chemical and energy consumptions were also determined for about 150 facilities. This study is unique in terms of the following aspects: (1) evaluation of wide spectrum of textile sub-sectors in a country, (2) involvement of about 150 industrial facilities, (3) on-site detailed investigations, (4) comparison of resource use performances of each facility and sub-sector with reference values given in TXT BREF, and (5) determination of potential reduction potentials for each sub-sector. We believe that this paper will provide valuable insights and useful data to legislators, researchers, textile facilities, and other stakeholders. Furthermore, this study will contribute to literature, as no published studies in this level of detail exist.

## Conclusions

The average specific water, energy, and chemical consumptions in Turkish textile sub-sectors were determined by making on-site industrial studies in about 150 facilities. Such consumptions were compared with reference values given in TXT BREF Document and potential reductions in consumptions were obtained. Thus, a very detailed picture/profile of cleaner production performance of textile sub-sectors was generated. The following textile sub-sectors were investigated in detail: cotton woven fabric dyeing-finishing, wool woven fabric dyeing-finishing, synthetic woven fabric dyeing-finishing, cotton knitted fabric, synthetic knit fabric dyeing-finishing, non-woven fabric, and dyeing-finishing of knitted fabric. Opportunities for sustainability in terms of decreasing resource utilization were elucidated in various textile sub-sectors.

The results overall indicated that specific water, chemical, and energy consumptions were generally compatible with reference values for most of the sub-sectors. However, significant levels of further reduction potentials, especially for electricity and steam consumptions, were found. For all the studied sub-sectors country-wide, the lowest and highest reduction potentials in resource uses were 18 ± 15 and 73 ± 13%, respectively, indicating that further investments are needed for cleaner production. Identifying and implementing the most appropriate facility-specific cleaner production techniques (good management practices, machine modification or replacement, process optimization, recovery and reuse, etc.) seemed to be critical for the textile sector. National and international regulations, environmental standards of global customers, market competition, and cost/profit issues are the major drivers for the sector in efficient use of resources. On the other hand, there might be several barriers such as technical, economic, managerial, financial, or political factors that hinder the adoption of cleaner production practices by facilities. Factors such as high initial investment costs, technological constraints, and the need for workforce training may pose barriers to the successful adoption of cleaner production practices. In addition, barriers to accessing financial capital, an inadequate incentive system, limited expertise and consultancy, concerns about market competition, business blindness, inadequate implementation willingness, inadequacy of infrastructure, and lack of monitoring can also be counted among the barriers.

Improving resource use efficiency in textile sub-sectors requires a multifactorial approach. In this respect, facilities, government institutions, industry associations, and universities have responsibilities. It is necessary to monitor the efficiency of resource use in the facilities, to evaluate the performances routinely, to develop the technical and technological infrastructure to eliminate inefficiencies, and to structure and effectively implement the cleaner production approach within the facility. Relevant state institutions should establish the legal infrastructure necessary for prioritizing and disseminating cleaner production in the sector. They should also effectively monitor the applications, develop the necessary incentive mechanisms, prepare sectoral guide documents, set targets for reducing resource use, prepare sectoral action plans, and develop sectoral expertise. We believe that this unique paper will provide useful information to legislators, researchers, textile facilities, and other stakeholders since it contains a wide range of specific data for various textile sub-sectors, which is not available in current literature.

## Data Availability

All data generated or analyzed during this study are included in this published article (and its supplementary information files).
